# Multi‐Omics Analysis Reveals Biomarkers That Contribute to Biological Age Rejuvenation in Response to Single‐Blinded Randomized Placebo‐Controlled Therapeutic Plasma Exchange

**DOI:** 10.1111/acel.70103

**Published:** 2025-05-27

**Authors:** Matias Fuentealba, Dobri Kiprov, Kevin Schneider, Wei‐Chieh Mu, Prasanna Ashok Kumaar, Herbert Kasler, Jordan B. Burton, Mark Watson, Heather Halaweh, Christina D. King, Zehra Stara Yüksel, Chelo Roska‐Pamaong, Birgit Schilling, Eric Verdin, David Furman

**Affiliations:** ^1^ Buck Institute for Research on Aging Novato California USA; ^2^ Global Apheresis Inc. Mill Valley California USA; ^3^ Circulate Seattle Washington USA

**Keywords:** aging, anti‐aging, human, inflammation

## Abstract

We conducted a randomized, placebo‐controlled trial to assess the safety and biological age (BA) effects of various therapeutic plasma exchange (TPE) regimens in healthy adults over 50. Participants received bi‐weekly TPE with or without intravenous immunoglobulin (IVIG), monthly TPE, or placebo. Randomization was based on entry date, and treatments were blinded to maintain objectivity. Primary objectives were to assess long‐term TPE safety and changes in biological clocks. Secondary goals included identifying optimal regimens. Exploratory analyses profiled baseline clinical features and longitudinal changes across the epigenome, proteome, metabolome, glycome, immune cytokines, iAge, and immune cell composition. We demonstrate in 42 individuals randomized to various treatment arms or placebo that long‐term TPE was found to be safe, with only two adverse events requiring discontinuation and one related to IVIG. TPE significantly improved biological age markers, with 15 epigenetic clocks showing rejuvenation compared to placebo (FDR < 0.05). Biweekly TPE combined with intravenous immunoglobulin (TPE‐IVIG) proved most effective, inducing coordinated cellular and molecular responses, reversing age‐related immune decline, and modulating proteins linked to chronic inflammation. Integrative analysis identified baseline biomarkers predictive of positive outcomes, suggesting TPE‐IVIG is particularly beneficial for individuals with poorer initial health status. This is the first multi‐omics study to examine various TPE modalities to slow epigenetic biologic clocks, which demonstrate biological age rejuvenation and the molecular features associated with this rejuvenation.

**Trial Registration:** Registered trial NCT06534450 on clinicaltrials.gov under the purview of the Diagnostic Investigational Review Board.

## Graphical abstract

Therapeutic plasma exchange safely reduces biological age by up to 2.6 years. Multi‐omics analysis reveals coordinated cellular and molecular responses associated with aging rejuvenation.

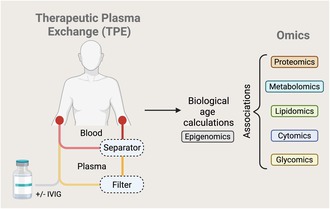

## 1 Introduction

Every country in the world is experiencing sustained growth in the proportion of older adults in its population. Current estimates predict that by 2030, almost a sixth of the world's population will be aged 60 years or older (Ageing and Health, n.d.). As age is the single most significant risk factor for chronic disease, these population dynamics pose a huge challenge for healthcare systems. However, a person's true age is much more than the chronological number of years they have lived. Factors like stress, diet, sleep, genetics, and infections influence the rate at which our bodies age, and biologically older individuals are more likely to develop diseases and experience premature mortality (Sigmond and Vellai 2023; Sayed et al. 2021; Meier et al. 2024; Waziry et al. 2019; Goeminne et al. 2024; Belsky et al. 2022; Furman et al. 2019). In recent years, we have witnessed an increasing number of studies focused on estimating a person's biological age (BA) at the molecular level based on changes to epigenomics (Bell et al. 2019), proteomics (Sayed et al. 2021; Johnson et al. 2020), metabolomics (Hertel et al. 2016), and other “omics” measurements (Rutledge et al. 2022; Alpert et al. 2019). The most accurate and well‐established methods utilize changes in DNA methylation (DNAm) patterns that occur with age, followed by machine learning techniques to construct biological age (BA) predictors. These BA “epigenetic clocks” are often predictive of diseases and mortality (Rutledge et al. 2022), and sensitive to various lifestyle interventions. For instance, moderate weight loss (< 5%) decreases epigenetic age by 1.1 years (Horvath clock), and individuals with more than 5% in weight loss show a decrease of 7.2 BA months in an 18‐months period (Yaskolka Meir et al. 2021). Nutrition also has a significant impact on methylation clocks. For example, methylation‐supportive diets combined with lifestyle changes can reduce biological age by 4.6 years in females (Fitzgerald et al. 2023) and 1.96 years in males (Fitzgerald et al. 2021). Healthy dietary habits maintained for up to 2 years can reduce age‐adjusted biological age by 0.66 years (GrimAge) (Fiorito et al. 2021). The effects of diet and nutritional supplementation seem to depend on the study population. For instance, in overweight African Americans, vitamin D3 supplementation can decrease epigenetic age by approximately 1.9 years (Chen et al. 2019) whereas in vitamin D‐deficient subjects, it decreases epigenetic age by only 1.3 years (Vetter et al. 2020; Vetter et al. 2022). Interestingly, a 60‐day relaxation practice scheme can also significantly impact epigenetic age with a rejuvenation of 4.67 years in healthy subjects (Pavanello et al. 2019). Pharmacological interventions have highlighted the potential of small molecules to reduce BA. For instance, a reduction in epigenetic age acceleration by 2.77 years (Horvath clock) and 3.43 years (Hannum clock) was observed in patients with diabetes mellitus undergoing metformin treatment (Li et al. 2022). Other pharmacological agents, such as dasatinib and quercetin (NCT04946383), or rapamycin (NCT04608448), are currently undergoing clinical trials targeting BA (Moqri et al. 2023). While several interventions have been associated with aging‐related biomarkers, their translation into consistent clinical benefit remains inconclusive, highlighting the need to distinguish between biomarker effects and validated clinical outcomes.

Methods to manipulate blood composition have proven promising in decreasing BA clocks and improving health status. For instance, intramuscular injection of human umbilical cord plasma concentrate into elderly human individuals has shown that youth factors substantially improve clinical biomarkers and reduce biological age by 0.82 years (using GrimAge) (Clement et al. 2022). Several studies have shown that therapeutic plasma exchange (TPE), first studied in animals in 1914 (Abel et al. 1914), can also significantly improve the outcomes of various medical indications. TPE, first used to treat macroglobulinemia in 1963 (Solomon and Fahey 1963) has recently been given Food and Drug Administration Emergency Use Authorization for the treatment of COVID‐19 (FDA, n.d.). Strikingly, up to 65% of patients with long COVID‐19 have shown improved peripheral neuropathy, fatigue, stamina, and brain fog after TPE treatment Kiprov, (2023). Interestingly, in cases of yellow phosphorus poisoning, TPE showed strong beneficial effects such as removal of the poison and improved liver function with associated changes in secreted circulating proteins and metabolites (Radhakrishnan et al. 2023).

In the context of aging, we recently conducted a study of eight individuals, demonstrating a remodeling of the immune system in the blood of older individuals and a decrease in proteins associated with aging after over five repeated TPE treatments (Kim et al. 2022). Expanding on our previous findings, this study applies a multi‐omics systems biology approach to longitudinally profile 30 individuals who underwent three therapeutic plasma exchange (TPE) modalities (10 individuals each): monthly TPE, biweekly TPE, and biweekly TPE combined with intravenous immunoglobulin (IVIG). We utilized BA deceleration as the primary endpoint by measuring 35 independent epigenetic clocks and estimated the BA rejuvenation effects caused by each intervention. Integrative analysis encompassing lipidomics, proteomics, metabolomics, cytomics, iAge (Sayed et al. 2021), the immune cytokine surrogate percentiles of iAge, and glycomics identified biomarkers in each ‘omics’ that are correlated with the responses to TPE and baseline clinical and ‘omics’ features that are predictive of the rejuvenation response to TPE treatment.

## 2 Results

**FIGURE 1:**
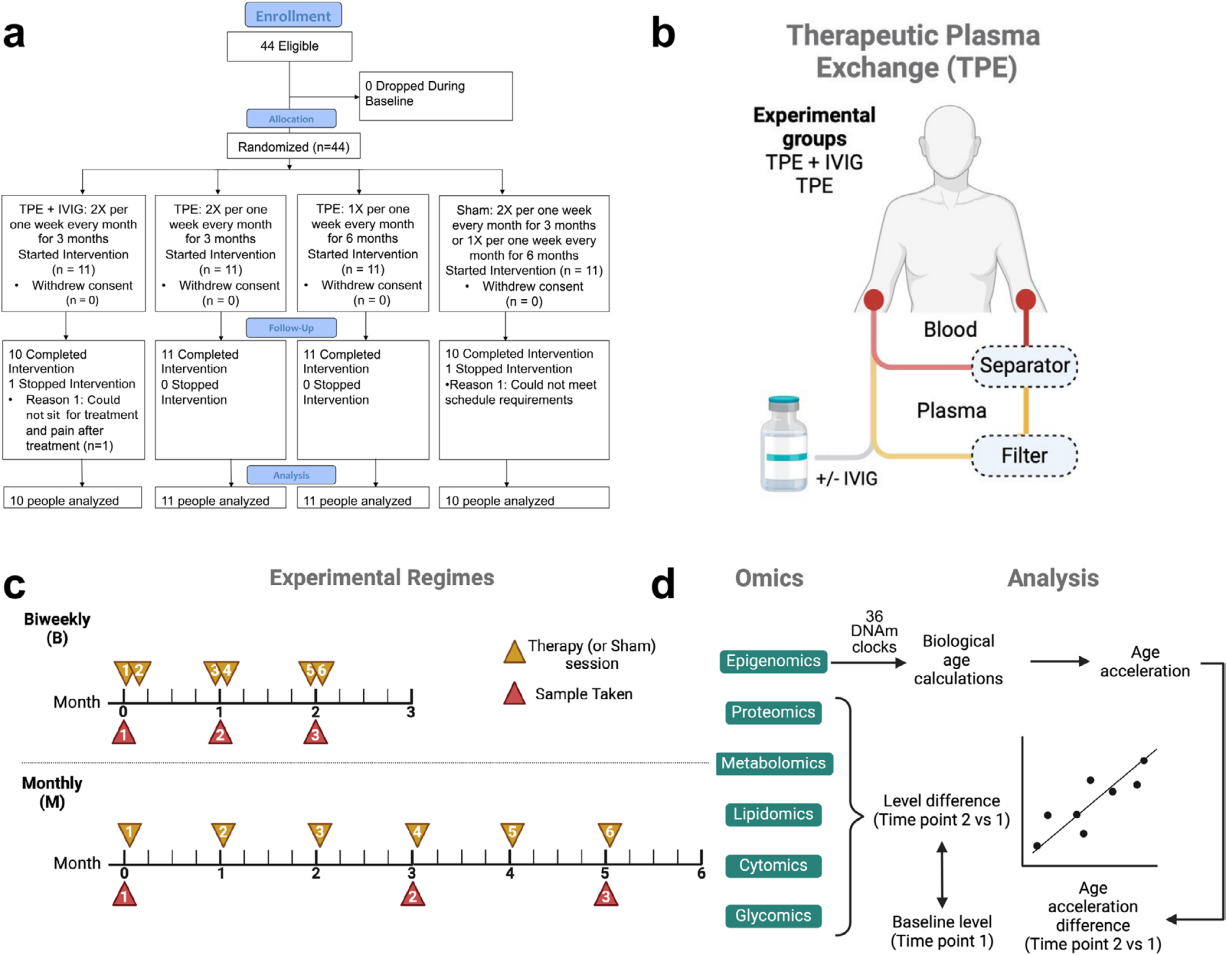
TPE study design. (a) Consort diagram for the trial. (b) Schematic representation of TPE treatment. In TPE, blood cells and plasma are separated. Plasma is then filtered and replaced with IVIG or fluids, and together with blood cells are returned to the patient's circulation. (c) Two temporal regimes were tested, with three blood samples taken per session. (d) Samples were used to perform multi‐omics profiling. Epigenomics data were used to calculate the differences in BA induced by the treatments. Correlation analyses were performed, comparing baseline and changing levels of the omics features and BA differences.

This clinical trial, categorized as a “Not Applicable” phase, involves healthy participants and utilizes TPE as an intervention (e.g., a device or behavioral approach) that was not intended for the diagnosis or treatment of any disease. Of the healthy individuals that expressed interest in this clinical trial to understand long‐term TPE safety and effect on epigenetic biological clocks, we enrolled 44 people under four different groupings (Figure 1). All the participants that began the trial were randomized and started the intervention (Figure 1a,b) of which 42 completed the study (Figure 1c). Table 1 presents sex and age characteristics for the four groups, with additional baseline measurements given in Table 2 and Supplemental Table S1.

**TABLE 1:** Age and sex characteristics of each cohort.

	Weekly TPE + IVIG	Weekly TPE	Sham	Monthly TPE
Average age	66.8	64.8	67.9	61.8
# of female	5	6	3	5
# of male	5	4	8	5
Average age female	66.5	65.1	77.0	61.8
Average age male	67.1	64.2	64.5	61.8

**TABLE 2:** Baseline epigenetic biologic clock acceleration of each cohort.

Clock	Bi‐weekly TPE + IVIG	Bi‐weekly TPE	Sham	Monthly TPE
AdaptAge	−3.19 (6.69)	2.44 (8.71)	−2.21 (14.39)	2.18 (5.59)
Blood	−2.04 (7.3)	−1.46 (7.76)	−2.37 (14.76)	5.16 (10.12)
Brain	−1.42 (11.9)	−2.85 (10.46)	−3.94 (17.49)	7.91 (15.53)
cAge	1.12 (2.84)	0.38 (3.77)	−2.02 (4.58)	0.38 (3.72)
CausAge	−0.15 (3.48)	−0.61 (4.53)	1.76 (10.61)	−0.09 (4.73)
DamAge	1.02 (7.03)	−1.01 (6.28)	3.6 (10.2)	−0.49 (4.18)
DNAmFitAge	0.4 (2.45)	0.4 (3.14)	−0.17 (4.33)	0.16 (4.44)
DNAmGait	−0.01 (0.07)	−0.04 (0.1)	0.08 (0.19)	0.01 (0.09)
DNAmGrip	0.68 (7.13)	−0.34 (6.79)	2.63 (6.18)	2.54 (7.59)
DNAmVO2max	0.22 (1.91)	−0.43 (1.9)	0.61 (2.39)	0.19 (1.06)
Hannum	0.86 (3.59)	−0.78 (4.41)	−0.77 (3.48)	1.79 (3.89)
Heart	−0.94 (7.84)	−0.05 (13.23)	−1.23 (12.22)	1.68 (7.14)
Hormone	0.28 (3.75)	−2.83 (5.19)	−1.01 (4.41)	1.16 (6.56)
Horvath	1.18 (6.05)	−0.02 (8.22)	0.56 (7.9)	−0.27 (3.96)
Immune	−1.24 (17.08)	1.66 (17.58)	12.28 (28.13)	1.28 (7.1)
Intrinsic Capacity	−0.27 (1.68)	0.81 (1.52)	−0.08 (1.74)	−0.07 (2.38)
Inflammation	1.26 (9.53)	−1.23 (9.32)	−2.45 (4.55)	5.87 (10.08)
IntrinClock	1.56 (2.77)	0.23 (3.43)	−1.14 (6.67)	−0.42 (4.8)
Kidney	−0.5 (8.61)	−1.62 (11.82)	1.19 (5.74)	4.87 (7.79)
Liver	−1.08 (9.01)	0.05 (6.72)	−1.17 (11.04)	2.63 (10.96)
Lung	0.48 (9.27)	2.37 (8.69)	3.11 (9.53)	−3.01 (9.28)
Metabolic	−0.08 (8.18)	−2.64 (8.8)	−6.5 (11.56)	7.52 (13.31)
MusculoSkeletal	−2.24 (10.61)	−5.71 (12.56)	2.59 (10.85)	5.61 (7.3)
OMICmAge	0.71 (2.94)	−1.03 (3.26)	2.42 (3.98)	0.68 (4.04)
PCDNAmTL	0.03 (0.14)	0.02 (0.16)	−0.04 (0.13)	−0.04 (0.15)
PCGrimAge	−0.01 (2.13)	−0.15 (3.3)	0.55 (1.99)	0.67 (1.83)
PCHannum	0.4 (5.36)	−0.59 (5.01)	−1.1 (4.94)	1.24 (3.33)
PCHorvath1	0.36 (4.85)	−0.09 (5.49)	−0.93 (6.08)	0.9 (3.9)
PCHorvath2	0.21 (4.75)	−0.63 (5.58)	−1.11 (6.89)	0.87 (3.12)
PCPhenoAge	0.45 (5.32)	−1.13 (5.94)	0.46 (5.8)	2.66 (5.38)
PhenoAge	−0.48 (4.62)	2.15 (4.34)	−1.03 (5.95)	0.75 (4.3)
Retroclock	0.2 (3.34)	0.01 (2.61)	−0.96 (5.44)	−0.24 (4.44)
Stochastic.Horvath	2.45 (4.46)	0.47 (7.71)	−2.91 (8.45)	−1.17 (5.4)
Stochastic.PhenoAge	1.11 (5.77)	−0.5 (4.64)	−0.68 (7.23)	−1.23 (3.01)
Stochastic.Zhang	2.45 (4.5)	0.14 (4.15)	−0.51 (5.8)	0.34 (4.73)
SystemsAge	−0.54 (8.25)	−0.57 (12.84)	−1.25 (11.23)	1.79 (8.43)

### 2.1 Compositional Changes of Epigenetic Biological Aging After TPE Treatment

**FIGURE 2:**
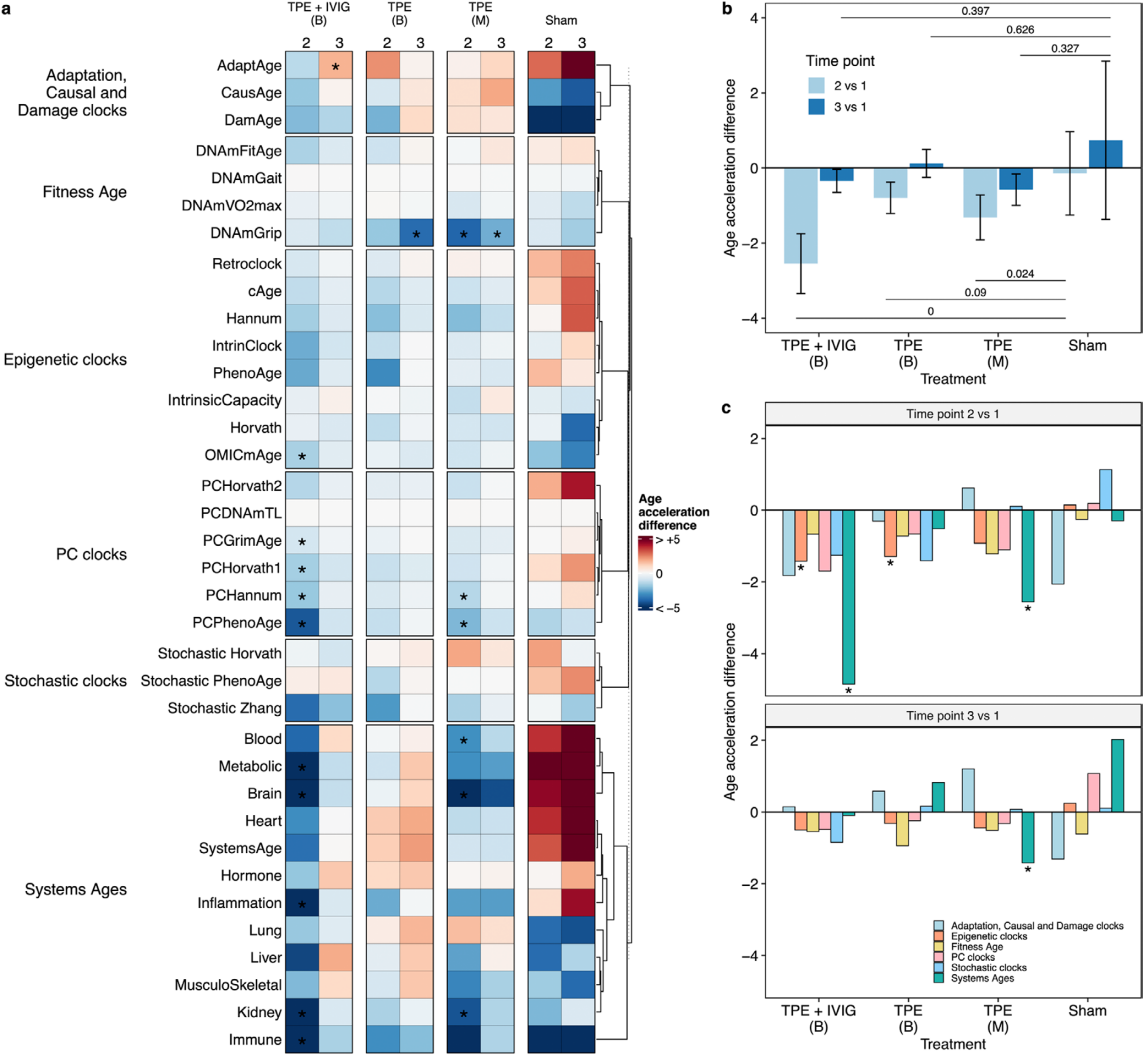
Therapeutic plasma exchange induces a coordinated biological age deceleration. (a) Biological age estimations using 36 epigenetic clocks. In all groups, we calculated the difference between the age‐adjusted biological age at time points 2 and 3 compared to time point 1 (age acceleration difference). No differences within groups (FDR corrected for 36 biologic clocks) were significant when *p*‐values were adjusted for the epigenetic clock comparisons (asterisk indicate nominal *p* value < 0.05). TPE + IVIG biweekly treatments has the most significant differences with Sham (Wilcoxon exact FDR < 0.05 after correction for 36 biologic clocks for each group) than other treatments (Table 4). (b) Average age acceleration difference across epigenetic clocks. Error bars indicate the 95% confidence intervals of the mean. FDR at the top and bottom indicate the significance of the difference between the treatments and sham using a Wilcoxon exact test. (c) Average age acceleration difference for groups of epigenetic clocks. Asterisks indicate age acceleration differences significantly different from zero (Wilcoxon exact FDR < 0.05).

Comparison of baseline measurements indicates that most of the biologic clocks significantly differ between the groups (FDR < 0.05 in Supplemental Table S2) and other baseline characteristics are similar between groups (except for ALDOA and VCAM1 in TPE + IVIG (B) vs. TPE (M)). No biologic clocks had a significant change from baseline to time point 2 or baseline to time point 3 with FDR correction; however, due to the low sample size and expected higher variance in human biological samples, we used nominal *p* values to identify increasing and decreasing trends in eight epigenetic biological clocks within groups between time point 2 and baseline and time point 3 and baseline (nominal *p*‐value < 0.05 in Figure 2a and Table 3). There were no clinically meaningful changes (e.g., functional, cognitive, or symptomatic) observed or assessed in this short‐term study. Significant differences between active treatment groups and Sham were identified in ten biologic clocks in TPE + IVIG (B), five biologic clocks in TPE (B), and five biologic clocks in TPE (M) (FDR < 0.05 in Table 4; corrected for 36 biologic clocks). Due to most biologic clocks differing at baseline, we confirmed that the changes were identified in time point 2 from baseline in each group were not due to significant intra‐individual variability for each clock and group (Table 5).

**TABLE 3:** Epigenetic biologic clock changes after TPE at time point 2.

Clock	Bi‐weekly TPE + IVIG	Within group *p* value*,^+^	Bi‐weekly TPE	Within group *p* value*	Sham	Within group *p* value*	Monthly TPE	Within group *p* value*,^+^
AdaptAge	−1.35 (9.68)	0.77	2.31 (5.95)	0.69	2.87 (17.67)	0.38	0.23 (2.77)	0.77
Blood	−3.94 (8.55)	0.19	−0.12 (2.73)	0.81	3.73 (20.09)	0.38	−3.07 (4.27)	0.05
Brain	−6.87 (10.75)	0.05	−0.29 (6.39)	0.69	4.44 (26.11)	0.92	−5.42 (6.66)	0.04
cAge	−1.27 (5.03)	1.00	−1.42 (1.65)	0.08	1.15 (5.59)	0.56	−1.07 (2.55)	0.43
CausAge	−1.91 (5.27)	0.43	−0.85 (1.52)	0.16	−2.84 (15.33)	1.00	0.83 (3.43)	0.38
DamAge	−2.2 (4.18)	0.13	−2.38 (5.06)	0.22	−6.21 (17.27)	0.28	0.8 (4.65)	0.16
DNAmFitAge	−1.58 (4.17)	0.49	−1.05 (2.46)	0.58	0.41 (1.99)	0.63	−0.07 (1.47)	0.85
DNAmGait	0.02 (0.07)	0.38	0.01 (0.07)	0.81	−0.08 (0.23)	0.70	0.01 (0.04)	1.00
DNAmGrip	−0.72 (5.41)	0.13	−1.91 (4.33)	0.22	−0.75 (5.33)	0.70	−3.99 (5.66)	0.00 (0.07)
DNAmVO2max	−0.39 (1.16)	0.11	0.05 (2.09)	0.94	−0.61 (2.94)	0.77	−0.81 (1.18)	0.06
Hannum	−1.72 (5.04)	0.38	−2.11 (2.84)	0.11	0.11 (3.35)	0.92	−2.18 (3.77)	0.16
Heart	−3.14 (6.66)	0.28	1.29 (6.7)	0.94	3.8 (12.14)	0.43	−1.31 (4.97)	0.38
Hormone	−1.95 (5.85)	0.43	0.97 (2.78)	0.47	0.09 (1.99)	1.00	0.08 (3.35)	0.85
Horvath	−0.39 (3.26)	0.92	−1.28 (4.38)	0.30	−0.32 (6.16)	0.70	−0.92 (3.58)	0.70
Immune	−9.77 (10.33)	0.01 (0.21)	−3.1 (8.19)	0.30	−13.13 (37.03)	0.23	−5.39 (11.42)	0.16
Intrinsic Capacity	−0.70 (1.69)	0.03 (0.90)	0.75 (1.03)	0.22	−0.61 (1.73)	0.70	−1.29 (3.63)	0.38
Inflamma‐tion	−7.18 (9.49)	0.19	−2.52 (4.45)	0.22	0.85 (6.62)	0.43	−2.76 (5.31)	0.43
IntrinClock	−2.49 (5.91)	0.49	−1.2 (2.4)	0.94	−0.46 (7.29)	0.63	−0.71 (2.48)	0.13
Kidney	−6.2 (8.12)	0.04	−1.61 (4.97)	0.47	−2.27 (8.07)	0.56	−4.33 (7.57)	0.04
Liver	−4.57 (8.1)	0.11	−0.53 (5.45)	0.94	−3.83 (11.31)	0.56	−2.7 (5.12)	0.16
Lung	−1.9 (4.64)	0.38	0.4 (4.83)	0.81	−3.87 (15.91)	1.00	1.55 (3.01)	0.19
Metabolic	−6.72 (9.71)	0.03 (0.90)	−1.06 (8.6)	0.81	5.2 (16.87)	0.56	−3.06 (5.08)	0.13
MusculoSkeletal	−2.22 (6.75)	0.43	−0.86 (6.47)	0.47	−1.79 (11.83)	0.92	−3.31 (7.89)	0.28
OMICmAge	−1.64 (2.12)	0.04	−0.35 (2.19)	0.94	−2.01 (4.98)	0.19	−1.01 (2.53)	0.19
PCDNAmTL	0.01 (0.07)	0.28	0 (0.05)	0.81	0.04 (0.08)	0.16	0.03 (0.06)	0.13
PCGrimAge	−0.9 (1.26)	0.05	−0.32 (1.36)	0.69	−0.01 (1.36)	1.00	−0.8 (1.73)	0.23
PCHannum	−1.89 (3.26)	0.05	−0.8 (2.11)	0.38	−0.06 (4.3)	1.00	−1.42 (3.36)	0.05
PCHorvath1	−1.69 (2.94)	0.01 (0.34)	−1.15 (1.95)	0.30	0.87 (5.01)	0.92	−1.02 (2.67)	0.28
PCHorvath2	−1.45 (3.65)	0.19	−0.54 (2.69)	0.81	1.83 (7.14)	0.85	−1.14 (2.77)	0.16
PCPhenoAge	−4.25 (5.86)	0.01 (0.46)	−1.18 (3.38)	0.47	−1.53 (6.74)	0.63	−2.27 (2.99)	0.04
PhenoAge	−2.56 (9.25)	0.56	−3.2 (2.65)	0.08	1.59 (6.18)	0.63	−0.49 (4.9)	1.00
Retroclock	−0.85 (4.81)	0.85	−0.7 (1.94)	0.69	1.62 (5.47)	0.23	0.24 (2.71)	1.00
Stochastic.Horvath	−0.25 (4.33)	0.70	0.1 (4.28)	1.00	2.06 (6.91)	0.49	1.95 (4.22)	0.32
Stochastic.PhenoAge	0.34 (2.24)	0.85	−1.44 (2.88)	0.16	1.45 (10.34)	0.70	−0.03 (2.56)	0.77
Stochastic.Zhang	−3.86 (5.14)	0.11	−2.88 (4.02)	0.11	−0.12 (5.25)	0.85	−1.61 (5.05)	0.28
SystemsAge	−3.75 (7.66)	0.28	1.22 (6.39)	0.81	3.19 (10.99)	0.49	−0.98 (4.15)	0.43

* Unadjusted Wilcoxon exact test.

^+^ FDR adjust for 36 epigenetic tests shown in parenthesis when less than 1.

**TABLE 4:** Between‐group *p* value of epigenetic biologic age after treatment.

Clock	Bi‐weekly TPE + IVIG vs. Bi‐weekly TPE	Bi‐weekly TPE + IVIG vs. Sham	Bi‐weekly TPE + IVIG vs. Monthly TPE	Bi‐weekly TPE vs. Sham	Bi‐weekly TPE vs. Monthly TPE	Monthly TPE vs. Sham
AdaptAge	1 (0.38)	0.69 (0)	1 (0.22)	1 (0)	1 (0.38)	0.69 (0)
Blood	0.22 (0)	1 (0.09)	1 (0.46)	1 (0.08)	0 (0)	0.3 (0)
Brain	0.07 (0)	0 (0)	1 (1)	1 (0.77)	0 (0)	0.01 (0)
cAge	0.02 (0)	1 (0.01)	1 (0.46)	0 (0)	1 (0.04)	0.05 (0)
CausAge	1 (0.56)	1 (0.01)	0.05 (0)	1 (0.04)	0.01 (0)	1 (0.81)
DamAge	1 (0.77)	1 (0.62)	0 (0)	1 (0.24)	0 (0)	0 (0)
DNAmFitAge	1 (0.77)	0.3 (0)	1 (0.46)	0.22 (0)	1 (0.04)	1 (0.22)
DNAmGait	1 (0.38)	1 (0.09)	1 (0.22)	1 (0.02)	1 (0.14)	1 (0.22)
DNAmGrip	1 (0.56)	1 (0.62)	0.02 (0)	1 (1)	1 (0.01)	1 (0.01)
DNAmVO2max	1 (0.08)	1 (0.09)	1 (0.05)	1 (0.04)	0.22 (0)	1 (0.03)
Hannum	1 (0)	1 (0.01)	1 (0.14)	0.01 (0)	1 (0.77)	0.05 (0)
Heart	1 (0)	0.05 (0)	1 (0.46)	1 (0.14)	1 (0.08)	1 (0)
Hormone	1 (0)	1 (0.05)	1 (0.05)	1 (0.02)	1 (0.14)	1 (0.81)
Horvath	1 (0.56)	1 (0.14)	1 (0.09)	1 (0.24)	1 (1)	1 (0.33)
Immune	1 (0)	0.02 (0)	0.69 (0)	1 (1)	1 (0.38)	1 (0.22)
Inflammation	1 (0)	0 (0)	1 (0.03)	0.22 (0)	1 (0.77)	0.02 (0)
IntrinClock	1 (0.56)	1 (0.33)	1 (0.01)	1 (0.14)	1 (0.24)	1 (0.62)
IntrinsicCapcity	1 (0.56)	1 (0.01)	1 (0.09)	1 (0.24)	1 (0.02)	0.02 (0)
Kidney	0.22 (0)	0.02 (0)	1 (0.09)	1 (0.24)	1 (0.04)	1 (0.01)
Liver	1 (0)	1 (0.14)	1 (0.81)	1 (0.14)	1 (0.04)	1 (0.81)
Lung	0.62 (0)	1 (0.14)	0 (0)	1 (0.14)	1 (0.77)	0.69 (0)
Metabolic	1 (0.08)	0.01 (0)	1 (0.03)	1 (0.04)	1 (0.56)	0.12 (0)
MusculoSkeletal	1 (0.56)	1 (0.14)	1 (0.33)	1 (0.56)	1 (0.38)	1 (0.09)
OMICmAge	0.22 (0)	1 (0.01)	0.05 (0)	1 (0.02)	1 (0.01)	1 (0.03)
PCDNAmTL	1 (1)	1 (0.81)	1 (0)	1 (0.24)	1 (0)	1 (0.46)
PCGrimAge	1 (0.04)	0.05 (0)	1 (0.33)	1 (0.14)	1 (1)	1 (0.01)
PCHannum	1 (0.04)	0.02 (0)	0.69 (0)	1 (0.14)	1 (0.77)	1 (0)
PCHorvath1	1 (1)	0.12 (0)	0.12 (0)	1 (0.04)	1 (0.04)	1 (0.22)
PCHorvath2	1 (0.38)	1 (0.09)	1 (0.81)	1 (0.04)	1 (1)	1 (0.22)
PCPhenoAge	1 (0.01)	1 (0)	1 (0.03)	1 (0.77)	1 (0.56)	1 (0.14)
PhenoAge	1 (0.08)	0.3 (0)	1 (0.81)	0 (0)	0.01 (0)	1 (0.09)
Retroclock	1 (0.04)	0 (0)	1 (0.33)	0 (0)	0.22 (0)	0 (0)
Stochastic Horvath	1 (0.56)	1 (0.05)	0 (0)	1 (0.24)	1 (0.02)	1 (0.81)
Stochastic PhenoAge	0 (0)	0.3 (0)	1 (0.14)	1 (0.38)	0.62 (0)	0.69 (0)
Stochastic Zhang	1 (0.77)	0.05 (0)	1 (0.09)	0.02 (0)	1 (0.01)	1 (0.01)
SystemsAge	1 (0.01)	0.05 (0)	1 (0.33)	1 (0.77)	1 (0.04)	1 (0)

*Note:* FDR corrected *p* values given with nominal *p* values in parentheses for Wilcoxon exact test. FDR < 0.5 highlighted in orange.

**TABLE 5:** Test of intra‐individual variability.

Clock	Levene test for Group TPE + IVIG (B), TPE (B) and TPE (M)
AdaptAge	0.97
Blood	0.86
Brain	0.98
cAge	0.74
CausAge	0.90
DamAge	0.79
DNAmFitAge	0.65
DNAmGait	0.71
DNAmGrip	0.24
DNAmVO2max	0.98
Hannum	0.21
Heart	0.32
Hormone	0.93
Horvath	0.13
Immune	0.38
Intrinsic Capacity	0.31
Inflammation	0.94
IntrinClock	0.54
Kidney	0.67
Liver	0.68
Lung	0.94
Metabolic	0.85
MusculoSkeletal	0.69
OMICmAge	0.31
PCDNAmTL	0.59
PCGrimAge	0.49
PCHannum	0.77
PCHorvath1	0.38
PCHorvath2	0.58
PCPhenoAge	0.76
PhenoAge	0.58
Retroclock	0.58
Stochastic.Horvath	0.70
Stochastic.PhenoAge	0.63
Stochastic.Zhang	0.60
SystemsAge	0.79

### 2.2 TPE Safety

Long term use of TPE was determined to be safe based on having a single mild allergic reaction in 240 procedures (0.42%). [Correction added on 17 June 2025, after first online publication: Section 2.2 is replaced in this version.]

### 2.3 TPE Induces Biological Age Rejuvenation and IVIG Supplementation Enhances the Effect

Subjects underwent therapeutic plasma exchange in two temporal regimes. Eleven individuals were subjected to two sessions during the first week, followed by 3 weeks with no sessions for 3 months (TPE biweekly regime), and 11 individuals received one session per month 6 times (TPE monthly regime) (Figure 1c). Blood samples were taken in both regimes before sessions 1 (baseline), 4, and 6. In addition to TPE, we evaluated in 10 individuals the effects of biweekly intravenous injections of immunoglobulin (IVIG) supplementation, which has previously been shown to enhance the immune system's ability to fight infections (Megha and Mohanan 2021; Katragkou et al. 2018). The control group received sham plasma exchange in either the biweekly or monthly regime, which mimicked the look and feel of TPE but without separation, filtration, or fluid replacement (see Methods).

We collected epigenetic data from the treated subjects and calculated DNA methylation age using 36 different epigenetic clocks. We adjusted the biological ages for each clock by the chronological age and for unknown covariates using the first three principal components of the control probes (see Methods), resulting in a metric of age acceleration. This metric represents the deviation of BA from individuals of the same age. To estimate the effects on BA induced by TPE, TPE, and IVIG, and Sham, we calculated the difference between the age acceleration from time point 1 to time points 2 or from time point 1 to time point 3. This age acceleration difference was negative if the interventions reduced biological age and positive if the interventions increased biological age, independently of age (Table 3, Figure 2a). We observed that at time point 2, all interventions induced a negative age acceleration difference compared to sham (Wilcoxon exact FDR < 0.05; Figure 2b). TPE + IVIG treatment displayed the largest reduction in BA, with an average decrease of 2.61 years (FDR = 6.22e‐05). In the case of TPE, the monthly regime showed a greater decrease in biological age than the biweekly regime, with an average BA rejuvenation of 1.32 years (FDR = 2.42e‐02), suggesting that more frequent sessions do not necessarily lead to a greater BA rejuvenation effect. Surprisingly, we observed no significant BA differences at time point 3 compared to sham in any group, suggesting potential compensatory mechanisms that mitigate the anti‐aging effects after multiple sessions.

Given that epigenetic clocks capture different aspects of aging, we asked which type of BA clock had the greatest effect. To do so, we grouped these clocks into six different types, depending on the method and outcome regressed. At time point 2, Systems age clocks (Sehgal et al. 2024) showed the largest decrease in age‐adjusted BA in the TPE + IVIG group (4.85 years, Wilcoxon exact versus zero FDR = 2.93e‐03 corrected for six clock groups and two comparisons for each TPE treatment group; 36 comparisons) and the monthly TPE intervention (2.55 years, FDR = 2.93e‐02) but not in the TPE biweekly group.

(FDR = 1) (Figure 2c). No biologic clocks in the Sham group had any significant change versus zero (FDR = 1). The non‐categorized epigenetic clocks (Epigenetic clocks in Figure 2a) also showed significant age acceleration difference in the TPE + IVIG (FDR = 3.91e‐02) and TPE (FDR = 4.69e‐02) interventions under a biweekly regime. Consistent with the average calculations across epigenetic clocks, most clock groups showed a reduced BA rejuvenation at time point 3. Given the attenuated effect at time point 3, we wondered if it was dependent on the effects observed at the previous session (time point 2), which could indicate a potential compensatory mechanism. Only bi‐weekly TPE interventions had a significant negative correlation (Spearman correlation and nominal permuted Spearman's correlation test) between the age acceleration differences at time point 2 from baseline and those between time point 3 and 2 (*p* < 0.048). The Spearman correlation in bi‐weekly TPE + IVIG treatment trends was negative but may be driven by the few samples in the analysis and is not significant. The sham group showed no such correlation. Overall, these results indicate that individuals with TPE had most of the rejuvenation effects between the first two collection points.

### 2.4 Omics Changes Linked to Rejuvenation in TPE + IVIG

To provide a better understanding of the molecular basis of the BA rejuvenation observed, we performed multi‐omics profiling on the same samples, including cytomics, glycomics, lipidomics, metabolomics, and proteomics (see Methods). To identify omics markers associated with the rejuvenation effects observed in time point 2, we calculated the correlations between the age acceleration differences induced by each intervention and the change in levels of each feature for each omics. We focused on features whose correlation was significantly different from zero and the sham intervention with an FDR < 0.05 (Supplementary Table 3, see Methods).

**FIGURE 3:**
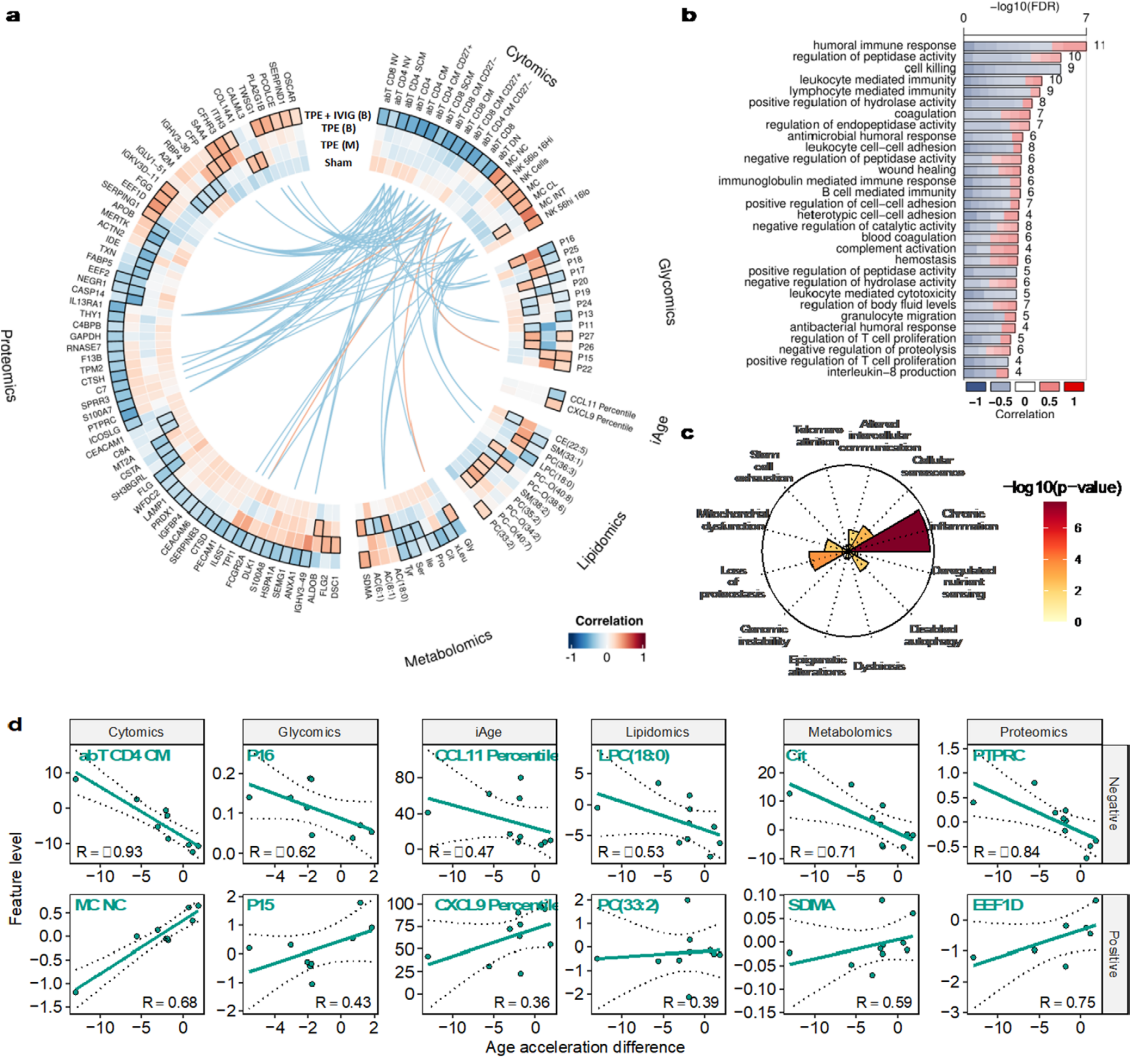
Multiple ‘omics’ features and cellular senescence proteins track with biological age rejuvenation response to TPE‐IVIG. (a) Correlations between the rejuvenation effects and changes in levels of omics features. We only display features significantly different from zero and sham in the TPE + IVIG group (permuted *t*‐test of the Fisherz adjusted Spearman correlations FDR < 0.05). Lines between the features at the center indicate inter‐feature correlations between cell composition changes and other omics (with data in at least eight individuals with a Spearman |R| > 0.8 and a permuted Spearman correlation test with a nominal *p*‐value < 0.05). (b) Gene ontology enrichment analysis of proteins with levels correlated with the responses to TPE + IVIG indicate activation of the immune system. (c) Enrichment of hallmarks of aging gene sets among proteins significantly associated with the TPE + IVIG response. (d) Features with the most robust positive and negative correlation with the rejuvenation effects in TPE + IVIG (with data in at least eight individuals and with FDR against zero < 0.05 for all comparisons with a nominal *p* value).

Overall, we observed that TPE + IVIG induced the most changes in omics profiles covering 83 of the 124 features affected by all interventions (Figure 3a). Notably, 95% of the cell types measured in the cytomics changed in proportion in coordination with the biological age differences after TPE + IVIG treatment. In contrast, we observed minor or no changes in cell types in TPE interventions, indicating that IVIG induced major changes in cell type composition, some of which could potentially contribute to the rejuvenation effects of BA. For example, we found that the BA rejuvenation effects were associated with a higher proportion of CD8^+^ and CD4^+^ naive T cells, a hallmark of immune aging, and lower levels of NK cells and monocytes. In addition, TPE intervention that decreases BA is correlated with a decrease in CXCL9 percentile, a surrogate of iAge positively correlated with a decrease in cardiovascular health (Sayed et al. 2021). These results are consistent with previous observations that CD8^+^ and CD4^+^ naive T cells significantly drop with age, while NK cells and monocytes (MC in Figure 3a) tend to increase with age (Egorov et al. 2018; Ligotti et al. 2021). Similarly, in proteomics, 43 proteins, representing 16.02% of the proteome measured in this study, displayed significant correlations with the BA changes in TPE + IVIG, compared to around 3% or less for the other TPE groups. To provide a better understanding of the function of these proteins, we performed gene ontology enrichment analysis. Proteins with levels correlated with rejuvenation effects in TPE + IVIG were mostly involved in the activation of the immune response, T cell proliferation, and cell–cell adhesion (Figure 3b). We also analyzed whether these proteins were associated with the hallmarks of aging. To do so, we compared these against precalculated sets of genes linked with the hallmarks of aging (see Methods). We observed a significant enrichment of proteins involved in altered chronic inflammation (FDR = 3.11e‐07), cellular senescence (FDR = 1.90e‐02), and loss of proteostasis (FDR = 2.71e‐03) (Figure 3c).

Given the large cell type composition changes observed, we investigated if these were correlated with other omics features that were significantly changing (inter‐omics correlations) (Figure 3a, blue lines for negative correlations and red lines for positive correlations). Indeed, among the most robust features changing in each omics (Figure 3d), several were strongly correlated (Spearman |r| > 0.8) with cell type changes due to TPE + IVIG intervention. Variability in changes related to TPE intervention among individuals indicate the heterogeneity of biological responses within this small cohort. For instance, 45% (9/20) of the cell type changes linked with rejuvenation in TPE + IVIG showed a significant correlation with the level of the soluble receptor for interleukin‐13 (IL13RA1), a key Th2 cytokine with a major effect in fibrosis. Similarly, changes in the levels of glycine (Gly) were correlated with the changes in two cell types. Interestingly, glycine has been shown to act on a variety of inflammatory cells like macrophages to reduce the formation of free radicals and inflammatory cytokines throughout the modulation of the expression of nuclear factor kappa B (NF‐κB) (Aguayo‐Cerón et al. 2023). Interestingly, changes in iAge and its surrogates were not correlated with changes in any other omics. Overall, these results show that the modulation of cell type composition and proteomic changes associated with immunosenescence largely drive the biological age effects induced by TPE + IVIG intervention.

### 2.5 Baseline Measurements Correlated With Changes in Biological Age Acceleration From TPE + IVIG

**FIGURE 4:**
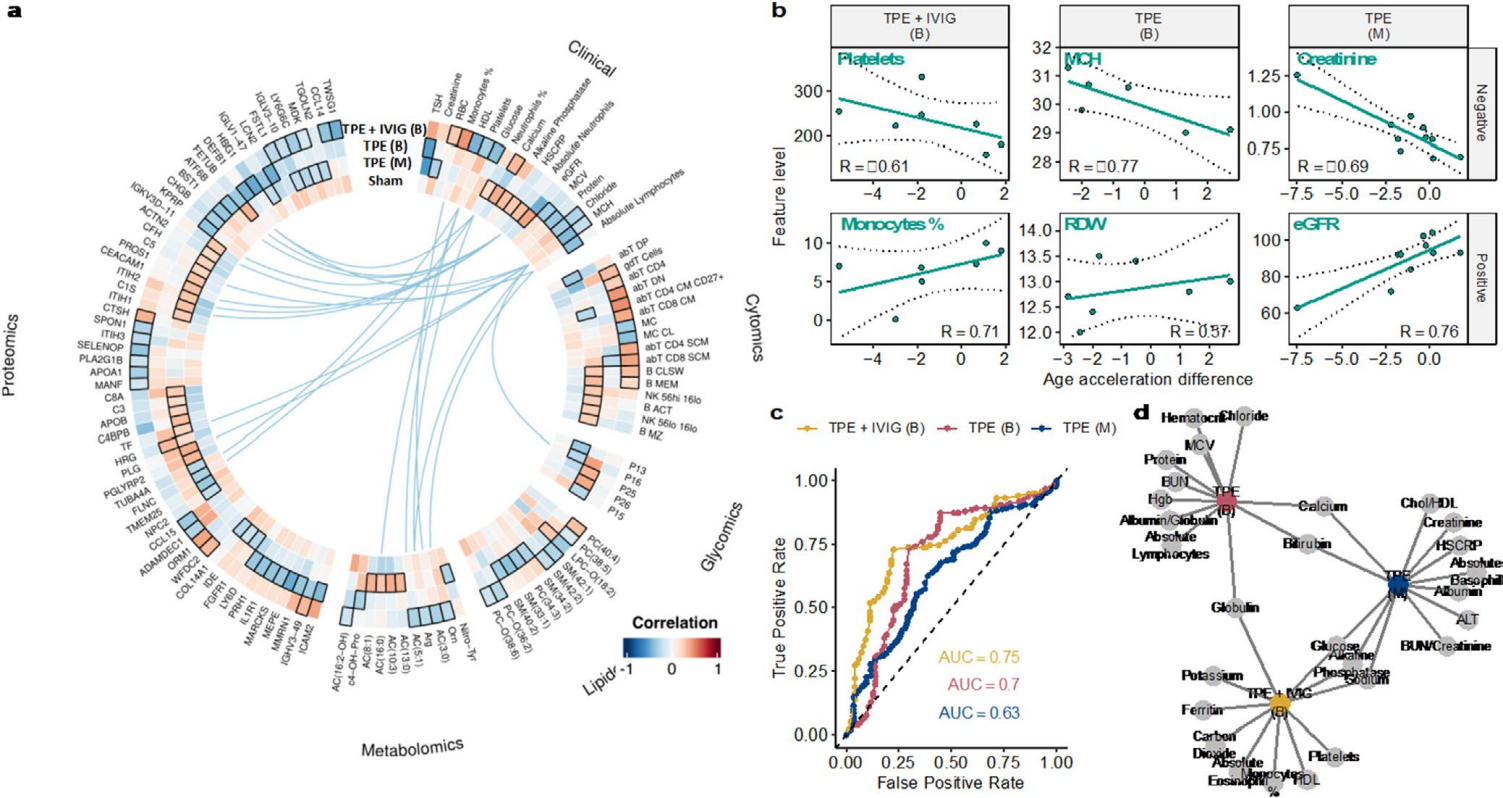
Baseline ‘omics’ and clinical biomarkers predict response to TPE‐IVIG. (a) ‘Omics’ features and clinical baseline levels correlated with the biological age response to TPE. The lines connecting the features at the center indicate inter‐feature correlations between clinical markers and other omics. (b) Clinical markers significantly correlated with the rejuvenation effects of TPE interventions. (c) ROC curve for the classification of responders to TPE interventions based on clinical markers. (d) Coefficients selected for the classification of responders in the TPE interventions.

One important aspect of interventional studies is the ability to determine a priori which individuals will respond to treatment. In our study, this corresponds to individuals who displayed a decreased age acceleration after the TPE interventions. To answer this, we used omics data from individuals at baseline (before treatment) and asked whether the magnitude of the rejuvenation effects correlated with the levels of any clinical or omics markers prior to treatment. We evaluated the correlation between the age‐adjusted age acceleration difference at time point 2 and the levels of clinical and omics markers at baseline (Tables S1 and S4, Figure 4a). In addition to the omics markers, we evaluated 57 clinical markers, which are more accessible and practical to measure in a clinical setting. We found a significant correlation between 18 clinical markers and the age acceleration differences in at least one experimental group (permuted t‐test of the Fisher's z‐transformed Spearman correlation; FDR < 0.05). Baseline iAge and its surrogates were not correlated with BA changes from TPE intervention. We found that monocyte percentage and platelets show correlation with age acceleration difference in TPE + IVIG with the most robust correlation with mean corpuscular hemoglobin (MCH) and change with TPE bi‐weekly treatment (Figure 4b), with higher levels being associated with the largest rejuvenation effects. While individuals in this study were nominally healthy, higher platelet levels and lower monocyte percentage levels within a healthy range were correlated with improvement in BA. These correlated levels indicate that an individual in overall poorer health may not benefit more from this treatment modality than someone in better health. Similarly, elevated levels of MCH and creatinine and lower levels of red blood cell distribution width (RDW) and epidermal growth factor (eGFR) at baseline are observed in individuals with the largest rejuvenation effects in TPE bi‐weekly and monthly, respectively. We also compared the features that were predictive of the response to the interventions in classification models (Supplementary Table 5, Figure 4c). We found that baseline levels of 12 (TPE biweekly) to 13 (TPE monthly) clinical markers were useful for classifying responders (age acceleration difference < 0) from non‐responders with alkaline phosphatase, bilirubin, calcium, glucose, sodium, and globulin contributing to the prediction of response to at least two interventions (Figure 4d). Also, classification of responders to TPE + IVIG was higher (AUC = 0.75) than TPE bi‐weekly (AUC = 0.7) or monthly (AUC 0.63). Overall, this analysis suggests that individuals with poorer health may experience the most significant improvements and that baseline levels are useful in predicting the biological age response to TPE.

## 3 Discussion

In this study, we aimed to assess the safety of long‐term TPE and the efficacy of treatment on BA. As a secondary objective, identification of an optimal TPE regime was assessed. We acknowledge the limited sample size as a key constraint of this study; given the exploratory nature of the trial, findings should be interpreted as hypothesis‐generating rather than definitive, and warrant validation in larger, adequately powered studies. We performed an exploratory analysis of follow‐up omics (time point 2 and time point 3) to better understand TPE treatment effects and baseline clinical and omics data to better understand how these baseline levels affect TPE treatment associated with BA changes. Our findings demonstrate that multiple TPE treatments with bi‐weekly or monthly sessions were safe with few adverse events and that three sessions of TPE treatment are sufficient to reduce biological age by 1.32 years and that IVIG supplementation doubles the effect to 2.61 years. Previous studies have assessed biological age upon TPE treatment in humans and animal models using clinical markers (Li et al. 2018), pro‐inflammatory markers (Kim et al. 2022), and functional tests (Mehdipour et al. 2022; Mehdipour et al. 2021; Mehdipour et al. 2020). The heterogeneity of individual responses observed with TPE treatment in this small study should be interpreted with caution and validated in larger, more diverse cohorts. However, besides two ongoing clinical trials (ClinicalTrials.gov identifiers NCT05004220, NCT03353597), this is the first study in humans to report a decrease in biological age by TPE using well‐validated epigenetic metrics of biological age.

Among the epigenetic clocks we used to quantify biological age, we found that Systems age clocks (Sehgal et al. 2024) displayed the most sensitive measures to the treatments, with biological age of the inflammatory and immune system decreasing 7.1 and 9.7 years, respectively, after TPE + IVIG treatment. However, TPE bi‐weekly and monthly showed a much smaller effect, ranging from 2.5 to 5.3 years. This result was consistent with the exploratory analysis that TPE + IVIG, but not TPE alone, induces dramatic changes in immune cell composition and that many of the changes reversed are characteristic of immunosenescence. For example, CD4 and CD8 naïve T cells decrease significantly with age, contributing to a reduced ability of the immune system and increased susceptibility to infections, increasing upon TPE + IVIG treatment. In particular, the strongest cell type change accompanying biological age rejuvenation was an increase in the percentage of CD4 and CD8 stem cell memory cells (SMC), a rare subset of T cells with self‐renewing and pluripotent properties (stem cell‐like) that also retain immunologic memory (Gattinoni et al. 2017). Interestingly, these cells display a strong age‐related decrease (Heikkilä et al. 2022) and are negatively correlated with disease severity in infections (Vigano et al. 2015; Ribeiro et al. 2014; Mateus et al. 2015), suggesting that the increase upon TPE + IVIG therapy might induce immunological rejuvenation. Given that these cell type changes are not observed in the TPE treatment, it is plausible that these effects are solely due to IVIG supplementation. However, it is possible that the supportive effects of IVIG in immunosenescence (Eränkö et al. 2018) synergize or enhance the rejuvenation effects of TPE. Thus, further experiments measuring BA and cell type composition changes associated with IVIG therapy are needed to disentangle these effects. At the proteomics level, we found that the increase in CD4 and CD8 sub‐cell types associated with BA rejuvenation was strongly correlated with an increase in the abundance of proteins involved in host defense through complement activation (C7), immune signaling (IL13RA1), Protein Tyrosine Phosphatase Receptor Type C (PTPRC) and Immunoglobulin Kappa Variable 3D‐11 (IGKV3D‐11). These findings support the hypothesis that TPE + IVIG therapy reverses not only features of immunosenescence but also promotes a more resilient immune response by enhancing host defense mechanisms.

Finally, our study also shows that baseline clinical testing can help to stratify patients for improved treatment efficacy. Here, whereas all subjects in this study were generally healthy and had laboratory values within the normal range, individuals with levels indicative of poorer health status, such as high blood glucose and lower circulating calcium, may benefit more from TPE + IVIG treatment. This is consistent with observations that anti‐aging interventions have been shown to benefit unhealthy individuals the most (Dioum et al. 2022). This finding suggests that pre‐treatment clinical assessment may help in identifying personalized treatments more likely to induce biological age rejuvenation and that TPE + IVIG therapy has longer‐term positive therapy outcomes that might be particularly beneficial in individuals with suboptimal health.

## 4 Methods

### 4.1 Participants

The CONSORT diagram for enrollment in the trial is given in Figure 1. This “not applicable” phased clinical trial was a small single‐site randomized placebo‐controlled trial with the aim of measuring the safety of long term TPE and the effects on biomarkers and epigenetic biological clocks in healthy older individuals. As this was a feasibility trial primarily designed to assess biological signal, no formal power calculation was performed, and the sample size was not determined to detect statistically significant clinical outcomes. The number of individuals was chosen based on the safety profile evaluation in the primary objective. Eligible patients were men and women over 50 years of age, with the exception of one woman in her 40s, who had no known chronic clinical conditions. Exclusion criteria included poor peripheral vascular access, diagnosis of active malignancy or active infection, late‐stage Alzheimer's disease, symptomatic coronary artery disease, congestive heart failure, restrictive pulmonary disease, asthma, taking growth hormones, stem cells, stem cell products, having a psychiatric disorder or taking anti‐aging supplements, except for one patient (A4) who was taking rapamycin. A subset of patients had clinical blood tests completed at Quest Labs (Bay Area, CA) or Life Extension Foundation Labs (Fort Lauderdale, FL) prior to the start of the trial. The study protocol (NCT06534450) was approved by the Diagnostics Institutional Review Board (Cummaquid, MA). All participants provided written informed consent.

### 4.2 Treatment Assignment

Randomization was carried out based on the first‐come first‐served principle by Global Apheresis. Among the suitable candidates, those that completed their initial lab tests and were eligible to participate were scheduled for their treatments in the order that they applied. The shorter duration testing groups (biweekly TPE groups with and without IVIG) were filled, followed by longer duration testing groups (monthly TPE or placebo group). Patients, caregivers, and raters were blinded.

### 4.3 Study Design and Intervention

TPE was performed using a centrifugal blood separator (Spectra Optia, Lakewood, CO) as an outpatient procedure by Global Apheresis in California. During each procedure, one plasma volume was removed and replaced with 5% albumin. The patients in the TPE‐IVIG group received 2 g of IVIG immediately after the TPE procedure. Enrolled patients were randomly allocated to four groups (in a 1:1:1:1 scheme): three TPE treatment groups and one control group that underwent a simulated TPE treatment through a noninvasive procedure (sham) that mimicked TPE but without any actual fluid replacement, with the patient receiving approximately 250 cc of normal saline. The three TPE groups included a twice‐a‐week treatment once per month, a twice‐a‐week treatment once per month with IVIG added, and a once‐a‐week treatment once per month. Samples were taken before the first, fourth, and sixth TPE treatments. From a total of 240 TPE procedures, only 1 (0.42%) mild allergic reaction to albumin was observed as an adverse event for participants that completed the intervention. All samples collected at the single study site were processed and sent for analysis (see each analysis below).

Subjects in the Sham group had a peripheral vein IV inserted in each arm, similar to the actual treatment subjects, but with only normal saline IV fluid. Dark curtains were used to hide the apheresis device and the pumps from the participants' field of view. The apheresis device was turned on and ran using a container filled with water, with both the access and the return lines submerged in the container to simulate blood flow through the machine. The device was programmed using the patient's information (height, weight, etc.) and a rinseback procedure was done to maintain the perception that the actual TPE procedure was finished.

### 4.4 Primary and Secondary Outcomes

The primary objective is to assure the safety of long‐term TPE and its effects on biomarkers and epigenetic biologic clocks. Given the short duration of the study, functional, cognitive, or symptomatic outcomes were not assessed as predefined endpoints. Secondary objectives include determining which TPE treatment group is optimal and determining whether epigenetic changes occur after TPE.

### 4.5 Statistical Analysis

Briefly, changes in feature levels in each omics were calculated for time points 2 or 3 versus 1 and correlated against the age acceleration difference at time points 2 and 3. We only considered features measured in more than 3 samples. Correlations were transformed into z‐scores using Fisher transformation (May and Looney 2020) using custom scripts and compared against zero and sham using a t‐test. *p* values were adjusted for multiple tests in each omics using the Benjamini–Hochberg method (Benjamini and Hochberg 1995). Features with FDR < 0.01 against zero and sham were considered statistically significant. In the case of the comparison against baseline levels of clinical and omics markers, Wilcoxon exact tests (Hothorn et al. 2022) and permuted Spearman correlation tests (Garren 2017) were used to evaluate changes after treatment to the epigenetic biological clocks and other measured clinical and biological features.

### 4.6 Epigenomics

DNA methylation was evaluated using TruAge (developed by TruDiagnostic Inc., Lexington, KY). Peripheral whole blood samples were obtained and then mixed with a lysis buffer to preserve the cells. DNA extraction was performed, and 500 ng of DNA was subjected to bisulfite conversion using the EZ DNA Methylation Kit from Zymo Research, following the manufacturer's protocol. The bisulfite‐converted DNA samples were randomly allocated to designated Illumina Infinium EPIC850k Beadchip wells. The samples were amplified, hybridized onto the array, and subsequently stained. After washing steps, the variety was imaged using the Illumina iScan SQ instrument to capture raw image intensities, enabling further analysis. Raw IDAT files were processed using the minfi pipeline (Aryee et al. 2014). Low‐quality samples were detected using ENMix by examining the variance of internal controls and flagging those with values more than 3 standard deviations from the mean control probe value (Xu et al. 2021). However, no outlier samples were found, so all samples were included in the analysis. Single‐sample Noob (ssNoob) normalization was used in order to consistently normalize the samples across the multiple array types. The algorithms analyzed by TruAge include first‐ (Horvath and Hannum) and second‐ (phenoAge, systemsAge, OMICAge, and GrimAge) generation epigenetic clocks. Epigenetic age acceleration (EAA) of the first and second generation clocks was calculated as the residual of each clock regressed upon chronological age. To account for any perceived batch effects, the first three principal components calculated from the technical probes were calculated and used as adjustment factors when calculating the EAA.

Using the epigenetic age acceleration (EAA) calculated (see Methods—Epigenomics) we calculated the age acceleration difference as:

$$\mathrm{Age}\;\text{acceleration difference}\;\mathrm{at}\;\text{time point}\;x=\mathrm{EAA}\;\left(\text{time point}\;x\right)-\mathrm{EAA}\;\left(\text{time point}\;1\right).$$

The mean age acceleration difference across individuals was calculated for each of the 35 different epigenetic clocks in each group. The average age acceleration difference across epigenetic clocks was compared between treatment groups and sham using the Wilcoxon test.

### 4.7 Proteomics

All water, methanol, acetonitrile, formic acid, and trifluoracetic acid used were LC/MS grade. Perchloric acid (70%) was purchased from Sigma Aldrich (311421‐50ML). Sequencing grade modified trypsin was obtained from Promega (V511B). Indexed retention time standards (iRT peptides) were purchased from Biognosys. HLB columns (1 cc 10 mg) were purchased from Waters (186000383).

Plasma samples were thawed on ice, and two 125 μL aliquots per sample were transferred to 1.5 mL tubes. Samples were diluted 10‐fold with water and mixed with 70% perchloric acid to a final concentration of 3.5% (Winzler and Devor 1948; Zougman and Wiśniewski 2006; Viode et al. 2023). Samples were incubated at –20°C for 15 min and subsequently centrifuged at 3200 ×*g* for 60 min at 4°C. The supernatant was transferred to new tubes and acidified with 1% trifluoracetic acid. Perchloric acid was removed using HLB columns (10 mg, Waters). Columns were conditioned with 800 μL methanol and washed with 1600 μL of 0.1% trifluoracetic acid (TFA). The acidified supernatant was loaded onto the column, washed with 2400 μL of 0.1% TFA, and proteins were eluted with 800 μL of 0.1% TFA in 90% acetonitrile. Eluates were dried using a speed‐vac and resuspended in 50 μL of 0.5% sodium dodecyl sulfate in 100 mM triethylammonium bicarbonate (TEAB).

Proteins were denatured with 10% SDS at 90°C for 10 min, cooled to room temperature, and pH adjusted to ~7 using 1 M TEAB. Proteins were reduced with 250 mM dithiothreitol at 56°C for 10 min, alkylated with 250 mM iodoacetamide in the dark for 30 min, and acidified with 12% phosphoric acid. Proteins were trapped and digested on S‐Trap mini spin columns according to the manufacturer's instructions. Briefly, reduced and alkylated proteins were diluted with S‐Trap buffer, loaded onto the column, and digested with 4 μg trypsin in 50 mM TEAB in two stages—1 h at 47°C followed by an additional 4 μg trypsin and overnight incubation at 37°C. Peptides were eluted sequentially with 50 mM TEAB, 0.5% formic acid, and 0.5% formic acid in 50% acetonitrile. Eluates were pooled, dried using a speed‐vac, and resuspended in 1% formic acid. Peptides were desalted using HLB columns (10 mg, Waters). Columns were conditioned with 0.2% formic acid in 50% acetonitrile and washed with 0.2% formic acid. Samples were loaded, washed with 0.2% formic acid, and eluted with 0.2% formic acid in 50% acetonitrile. Eluates were dried using a speed‐vac and resuspended in 100 μL 0.2% formic acid. Samples were stored at −20°C until analysis. Prior to LC–MS/MS analysis, peptides were diluted 1:1 with 0.2% formic acid and spiked with 0.5 μL of iRT peptides (Biognosys) (Escher et al. 2012).

Reverse‐phase HPLC‐MS/MS data was acquired using a Waters M‐Class HPLC (Waters, Massachusetts, MA) connected to a ZenoTOF 7600 (SCIEX, Redwood City, CA) with an OptiFlow Turbo V Ion Source (SCIEX) equipped with a microelectrode (Burton et al. 2024). The chromatographic solvent system consisted of 0.1% formic acid in water (solvent A) and 99.9% acetonitrile, 0.1% formic acid in water (solvent B). Digested peptides (4 μL) were loaded onto a Luna Micro C18 trap column (20 × 0.30 mm, 5 μm particle size; Phenomenex, Torrance, CA) over a period of 2 min at a flow rate of 10 μL/min using 100% solvent A. Peptides were eluted onto a Kinetex XB‐C18 analytical column (15 × 0.30 mm, 2.6 μm particle size; Phenomenex) at a flow rate of 5 μL/min using a 120 min microflow gradient (as described below), with each gradient ranging from 5% to 32% solvent B. Briefly, 4 μL of digested peptides were loaded at 5% B and separated using a 120 min linear gradient from 5% to 32% B, followed by an increase to 80% B for 1 min, a hold at 80% B for 2 min, a decrease to 5% B for 1 min, and a hold at 5% B for 6 min. The total HPLC acquisition time was 130 min. The following MS parameters were used for all acquisitions: ion source gas 1 at 10 psi, ion source gas 2 at 25 psi, curtain gas at 30 psi, CAD gas at 7 psi, source temperature at 200°C, column temperature at 30°C, polarity set to positive, and spray voltage at 5000 V.

All human samples were acquired in data‐independent acquisition mode (DIA) analysis with two technical replicates for each biological sample replicate (Gillet et al. 2012; Collins et al. 2017; Schilling et al. 2017). Briefly, the DIA‐MS method on the ZenoTOF 7600 system is comprised of a survey MS1 scan (mass range: 395–1005 m/z), with an accumulation time of 100 ms, a declustering potential of 80 V, and a collision energy of 10 V. MS2 scans were acquired using 80 variable width windows across the precursor ion mass range (399.5–1000.5 m/z), with an MS2 accumulation time of 25 ms, dynamic collision energy enabled, charge state 2 selected, and Zeno pulsing enabled (total cycle time 2.5 s).

All data files were processed with Spectronaut v16 (version 16.0.220524.5300; Biognosys) performing a direct DIA search using the UniProt *Homo sapiens* reference proteome with 20,423 entries, accessed on 06/30/2023. Dynamic data extraction parameters and precision iRT calibration with local non‐linear regression were used. Trypsin/P was specified as the digestion enzyme, allowing for specific cleavages and up to two missed cleavages. Methionine oxidation and protein N‐terminus acetylation were set as dynamic modifications, while carbamidomethylation of cysteine was set as a static modification. Protein group identification (grouping for protein isomers) required at least two unique peptides and was performed using a 1% q‐value cutoff for both the precursor ion and protein level. Protein quantification was based on the peak areas of extracted ion chromatograms (XICs) of 3–6 MS2 fragment ions, specifically b‐ and y‐ions, with automatic normalization and 1% q‐value data filtering applied. Relative protein abundance changes were compared using the Storey method with paired t‐tests and *p* values corrected for multiple testing using group wise testing corrections (Burger 2018).

### 4.8 Glycomics

Glycan data was derived as described previously (Rapčan et al. 2024). For the analytical precision analysis, AMC, and long‐term variability, the IgG isolation, IgG *N*‐glycan release, and labeling were performed using a Genos‐Glycanage IgG glycome profiling kit (Genos, Osijek, Croatia) and subsequent capillary gel electrophoresis with laser‐induced fluorescence (CGE‐LIF) analysis was adapted from previously published protocols (Pučić et al. 2011; Hanić et al. 2019). The process of extracting IgG involved diluting 25 μL of subject plasma samples and three distinct plasma standards in quadruplicate, serving as technical replicates of a known, previously analyzed glycome. This dilution was carried out using a 1:7 ratio with a 1 × PBS buffer, which was prepared in‐house. Additionally, blank samples containing ultrapure water, without any analyte, were included to monitor and control for potential cross‐contamination. The diluted samples were resuspended and filtered through a wwPTFE filter plate with 0.45‐μm pore size (Pall corporation, New York, NY, USA) using a vacuum manifold and pump (Pall corporation, New York, NY, USA). The filtered samples were transferred to a CIM r‐Protein G LLD 0.05 mL monolithic 96‐well plate (Sartorius BIA Separations, Ajdovščina, Slovenia), where they underwent binding and subsequent washing steps with phosphate‐buffered saline (1 × PBS) buffer (0.25 M NaCl, increased ionic strength, prepared in‐house). Elution of the bound IgG was achieved by employing 0.1 M formic acid neutralized with ammonium bicarbonate buffer (Sigma‐Aldrich, St. Louis, MO, USA). The eluted IgG fraction (20 μL) was dried and prepared for the subsequent steps in the protocol.

The dried IgG samples were consecutively treated with 1.66 × PBS, 0.5% sodium dodecyl sulfate (SDS) and 2% Igepal (Sigma‐Aldrich, St. Louis, MO, USA/Invitrogen Thermo Fisher Scientific, Carlsbad, CA, USA) to denature the IgG, followed by incubation with 1.2 U of the enzyme PNGase F (Promega, Madison, WI, USA) at 37°C for 3 h to release its *N*‐glycans. The released glycans were then labeled by mixing APTS (8‐aminopyrene‐1,3,6‐trisulfonic acid) (Synchem, Felsberg, Germany) fluorescent dye with the reducing agent 2‐picoline borane (Sigma‐Aldrich, St. Louis, MO, USA) and subjected to a 16‐h incubation at 37°C.

After incubation, the labeling reaction was halted by the addition of 80% acetonitrile (ACN, Carlo Erba, Milan, Italy). The clean‐up of the released fluorescently labeled IgG *N*‐glycans was conducted using solid‐phase extraction utilizing Bio‐Gel P‐10 as a hydrophilic stationary phase. The entire sample volume was transferred to the filter plate containing the Bio‐Gel P‐10. The excess label and reducing agent were removed by five washes with 80% ACN/100 mM triethylamine (Sigma‐Aldrich, St. Louis, MO, USA), followed by three washes with 80% ACN. Finally, APTS labeled IgG *N*‐glycans were eluted in ultra‐pure water.

For CGE‐LIF analysis, 3 μL of purified IgG *N*‐glycans combined with 7 μL of Hi‐Di Formamide were analyzed using an ABI3500 Genetic Analyzer (Thermo Fisher Scientific, Waltham, MA, USA) equipped with a 50‐cm long 8‐capillary array filled with POP‐7 polymer as a separation matrix. Run parameters were set as follows: run time 1000 s, injection time 12 s, injection voltage 15 kV, run voltage 15 kV, and oven temperature 60°C. The resulting electropherograms were manually integrated into 27 glycan peaks using the Empower 3 software (Waters, Milford, MA, USA). The amount of glycan structures in a peak was expressed as a percentage of the total integrated area (total area normalization). In addition, six derived glycan traits were calculated for glycans with shared structural features (Trbojević Akmačić et al. 2015; Gornik et al. 2009).

### 4.9 Metabolomics and Lipidomics

Targeted metabolomics was performed on plasma samples utilizing the Biocrates AbsoluteIDQ p400 HR kit. Prior to analysis, a system suitability test and instrument calibration were performed with the QExactive mass spectrometer (Thermo Scientific). The experimental procedure involved processing plasma samples, blanks, calibration standards (7‐point), and quality controls according to the manufacturer's recommendations.

Specifically, 10 μL of plasma was added to a pre‐loaded filter plate containing internal standards and dried using ultra‐pure nitrogen. Subsequent derivatization with 5% phenylisothiocyanate in pyridine, ethanol, and water (in a 1:1:1 ratio), followed by extraction with 5 mM ammonium acetate in methanol, yielded extracts that were collected into a 96‐deep well plate via centrifugation. Mass spectrometric analysis was conducted using the Thermo QExactive mass spectrometer in positive ionization mode. Chromatographic separation utilized a proprietary Biocrates column, employing 0.2% formic acid in water as buffer A and 0.2% formic acid in acetonitrile as buffer B.

For the analysis of lipids, acylcarnitines, and hexoses, flow injection analysis (FIA) was employed without a column, utilizing the Biocrates FIA additive mobile phase. Data processing and lipid quantification were performed using Thermo Excalibur, QuanBrowser, and MetIDQ software. Normalization of peak areas corresponding to metabolites was conducted relative to their respective internal standards. Target metabolite concentrations were estimated linearly based on observed concentrations in quality control samples, and a seven‐point quadratic calibration approach was implemented where applicable. All chemicals and solvents used were LC/MS grade.

### 4.10 Cytomics

Blood was collected into lavender‐top vacutainer tubes (K‐EDTA, BD) and kept on ice until processing the same day. Peripheral blood mononuclear cells (PBMC) were prepared from the whole blood by density gradient centrifugation using Ficoll‐Paque PLUS (Cytivia) according to the package directions, followed by lysis of residual erythrocytes with ACK buffer (Gibco). After washing twice in dPBS, PBMC were cryopreserved in 90% fetal bovine serum (FBS) and 10% DMSO and stored in liquid nitrogen until analysis.

Vials of frozen PBMC (~2 million) were thawed rapidly by swirling in a 37°C water bath and immediately transferred to 15 mL conical tubes with 10 mL of pre‐warmed RPMI medium with 10% FBS, then pelleted for 5 min at 300 g in a benchtop TC centrifuge. After removal of the supernatant, the cells were resuspended in 4 mL of warmed RPMI/10% FBS and allowed to recover in a TC incubator for 3 h prior to staining. After recovery, the cells were divided equally for analysis with the SPiDERGal and intracellular reagent panels. For the SPiDERGal workflow, the cells were left in medium and treated with 1 μM Bafilomycin A1 (Abcam) for 1 h, followed by 667 nM of SPiDERGal (ThermoFisher) for one additional hour, while the cells for the IC panel were pelleted and kept on ice for staining. For both panels, the cells were resuspended in 200 μL of dPBS in V‐bottom plates and incubated for 20 min on ice with 1X Live/Dead Blue (ThermoFisher) plus 5 μg/mL of human IgG (Sigma). Surface marker‐specific antibodies plus FBS to 2% v/v were then added (as specified in the attached table), and the cells were incubated on ice in the dark for 1 h, then washed once in PBS/2% FBS. For the SPiDER panel, the cells were then taken up in 150 μL of PBS/2% FBS and acquired. For the IC panel, the cells were taken up in 150 μL of FOXP3 Fix/perm buffer (ThermoFisher) and incubated on ice for 20 min, pelleted, washed once with FOXP3 Perm‐wash buffer (ThermoFisher), and then stained with the IC marker‐specific antibodies in Perm‐wash buffer as specified in the attached table. They were then pelleted, washed once in Perm‐wash buffer, and resuspended in PBS/2% FBS for acquisition.

The stained PBMC were acquired on a 5‐laser Cytek Aurora spectral flow cytometer (Cytek), and the raw data were spectrally unmixed using the SpectroFlo software package (Cytek). Subsequent correction of spectral compensation and manual gating to identify populations were performed using FlowJo (software (BD)), and population frequencies (as % of parent pop and percent of live leukocytes) and numbers were tabulated for subsequent bioinformatic analysis.

### 4.11 iAge

Samples were analyzed using a Luminex LX‐200 instrument (Luminex Corp., Austin, TX, USA) to determine the levels of Inflammatory Age markers using Edifice's proprietary assay, composed of 5 core proteins: CCL11, IFN‐γ, GRO‐α, CXCL9, and TRAIL. Raw mean fluorescent intensity (MFI) values for each plate below the 5th percentile were set to the 5th percentile of the plate, and those above the 95th percentile were set to the 95th percentile of the plate. These values were normalized using control serum samples from 11 individuals spanning a diverse range of ages (23–83 years old) and both sexes. iAge was derived from all study participants using the Edifice proprietary machine learning algorithms using the normalized MFI values. The percentile is calculated for each individual from the empirical cumulative distribution of iAge in the study population from the same decade as the individual.

### 4.12 Bioinformatic Analysis

Circular and rectangular heatmaps were generated using the package circlize and ComplexHeatmap (Aradillas et al. 2015). Gene ontology enrichment analysis was performed using the function enrichGO implemented in the package clusterProfiler (Wu et al. 2021). Only GO biological process terms with a set size between 50 and 500 were analyzed. Enrichment results were plotted using the package CellPlot (dieterich‐lab/CellPlot 2024). To identify genes associated with each of the 12 hallmarks of aging, we used a corpus of 36 million abstracts from PubMed (https://huggingface.co/datasets/ncbi/pubmed). First, we identified 71,129 abstracts including the word “aging” or “ageing” in the title or abstract. Then, we used large language models (GPT‐4o mini) to analyze each abstract using the following query: “Your task is to identify genes associated with the hallmarks of aging from the following scientific abstract. For each gene mentioned in the abstract, annotate it with the corresponding hallmark of aging (genomic instability, telomere attrition, epigenetic alterations, loss of proteostasis, deregulated nutrient sensing, mitochondrial dysfunction, cellular senescence, stem cell exhaustion, altered intercellular communication, disabled autophagy, chronic inflammation, dysbiosis)”. To perform enrichment analysis using these gene sets, we ranked the genes in the query signature by −log10(*p* value) and then performed a one‐tailed gene set enrichment analysis using the package fgsea (Korotkevich et al. 2021). To build the classification models we only considered samples that measured at least 50% of the clinical markers and markers that were measured in at least 50% of the samples. Missing values were imputed using the R package impute. Each individual was classified into responder or non‐responder for each epigenetic clock based on the age acceleration difference (e.g., responder: age acceleration difference < 0, non‐responder: age acceleration difference > 0). We performed Elastic Net logistic regression on the clinical data using 10‐fold cross validation and we evaluated shrinkage parameter (lambda) of classification models using area under the curve. Intrinsic capacity was calculated from methylation data as previously described (Fuentealba et al. 2024). Intrinsic capacity acceleration was calculated the same as other epigenetic clocks (see Methods above) and multiplied by −100 to scale the value to a similar scale as other epigenetic clocks based on age.

## Author Contributions

D.F., D.K., and E.V. conceptualized the project. D.K., E.V., B.S., and D.F. provided supervision. D.F., M.F., K.S., P.A.K., H.K., J.B.B., W.‐C.M., M.W., C.D.K., Z.S.Y., C.R.‐P., and D.K. conducted the investigation. M.F., K.S., P.A.K., H.K., and J.B.B. performed formal data curation and analysis. M.F. and K.S. generated the visualizations. H.H. oversaw project administration. M.F., K.S., and D.F. drafted the original manuscript, and all authors edited the work and approved the final draft.

## Conflicts of Interest

D.K. (co‐founder and CSO), E.V., and D.F. are members of Circulate Inc. D.F. is the co‐founder of Edifice Health. All other authors declare no conflicts of interest.

## Supporting information


**Table S1.** Baseline clinical, iAge, proteomic, metabolomic, Lipidomic, glycomic, and cytomic measurements.

**Table S2.** Comparison of baseline measurements for clinical, iAge, proteomic, metabolomic, lipidomic, glycomic, and cytomic data between each treatment group.

**Table S3.** Correlation of the changes in clinical, iAge, proteomic, metabolomic, lipidomic, glycomic, and cytomic measurements and the changes in the epigenetic clocks at time point 2 versus time point 1.

**Table S4.** Correlation of baseline clinical, iAge, proteomic, metabolomic, lipidomic, glycomic, and cytomic measurements and the changes in the epigenetic clocks at time point 2 versus time point 1.

**Table S5.** Predictive baseline clinical, iAge, proteomic, metabolomic, lipidomic, glycomic, and cytomic measurements of response to treatment.

**Appendix S1.** CONSORT 2010 checklist of information to include when reporting a randomised trial.

## Data Availability

Processed data and code used to generate the figures can be found in https://github.com/msfuentealba/lyfspn. The raw data and complete mass spectrometry datasets have been uploaded to the Mass Spectrometry Interactive Virtual Environment (MassIVE) repository and can be downloaded using the following link: https://massive.ucsd.edu/ProteoSAFe/dataset.jsp?task=945e8acb15054ae895937c3b84d926b9 (MassIVE ID number: MSV000095275; ProteomeXchange ID: PXD053805).
